# On mountains and prophets: targeting majorities to support minorities by using norm-critics in health education

**DOI:** 10.1080/17482631.2018.1522203

**Published:** 2018-10-05

**Authors:** Britta Pelters

**Affiliations:** School of Health and Welfare, Halmstad University, Halmstad, Sweden

**Keywords:** Power, stigma, coping, norms, norm-critical education, health disparities

## Abstract

This debate article advocates for norm-critics instead of empowering coping and pedagogy of tolerance as an educational approach to mitigate stigmatization as well as blame and guilt for health-deviant minorities within the field of health disparities. Norm-critics is a way of making members of the (presumably healthy) normative majority uncover and question their health-related norms and raise awareness for the processes by which members of that majority re/construct images of stereotypic figures (such as “the fatso” or “the couch-potato”) with certain personal character traits which are to be condemned and, in doing so, limit the acting space of those identified as examples of those figures. The approach, its theoretical background, arguments promoting norm-critics, and some suggestions for its practical application are presented. It is concluded that norm-critics render a valuable and much needed addition to the health intervention repertoire.

## Introduction

What do people with overweight, a psychologic diagnosis, HIV/AIDS, or cancer have in common? They are all stigmatized and often feel excluded from the normative “healthy majority” of “common people” (see e.g., Barroso et al., 2014; Liu et al., 2016; Zäske & Gaebel, 2015), sometimes even as iatrogenic effects of public health communication interventions (Guttman & Salmon, 2004). It is moreover worth mentioning that not only the manifestly ill face blame, guilt, and stigmatization. Considering the preoccupation with and proactivity demanded of every individual in creating one’s health (Crawford, 1980) and processes of othering aiming at those who do not follow this demand by indulging in “the Holy Trinity of risk—to eat a lot, to be sedentary, to smoke” (Schmidt, 2010, p. 17), stigma, blame, and guilt concern a much bigger group of deviant minorities. That even more so as these practices are usually more common in those groups which health disparities research focuses on (Bartley, 2016; Fritzell & Lundberg, 2006).

What do interventions targeting these disadvantaged groups usually focus on in order to counteract their stigmatization? They may either focus on empowering these very groups to cope with stigma and counteract the negative consequences of stigmatization (e.g., Harper, Lemos, & Hosek, 2014) or present testimonies and information to potentially stigmatizing groups to increase knowledge and empathy for stigmatized groups (e.g., Peters et al., 2015). The latter delivers thus what Bromseth and Draj (2010) called “pedagogy of tolerance” (see also RFSL Ungdom and Forum för levande historia, 2011). As Guttman (2000) suggests, all interventions hold a normative perspective and are laden with assumptions and values regarding (among other things) the problem to be solved, the suggested solution, and the intervention bringing about this solution which needs to be known to understand their impact. This bears on the mentioned practised alternatives: whereas coping-focused interventions can be regarded representing a problem of understanding stigma on the part of the Othered group, pedagogy of tolerance deals with a problem of understanding the stigmatized (both intellectually and emotionally) on the part of the normative majority. On this basis, three major problematic implicit features of pedagogy of tolerance need to be highlighted:

- Achieving understanding by means of stereotyping: A certain group of “stigmatized people” can be comprehensively described to promote the aimed-for understanding. That effort not only describes but also defines the group. It elicits an image of “the stigmatized” as “Other”. Othering as the process leading to the image of “the Other” describes a constant (re)production of stereotypes as shared images that delineate ways of thinking and acting which are considered “abnormal”. Here, the Other is the unhealthy or the health-deviant as a member of societal groups that are “marginalized, denigrated or violated (i.e., Othered) in society” (Kumashiro, 2002, p. 32). The image of the Other will necessarily always be stereotypic (see also Crawford, 1994) as the diversity within the group needs to be decreased to be able to deliver a somehow coherent picture with the capacity to elicit understanding through knowledge and empathy to close the understanding gap which has been identified as “the problem”. Pedagogy of tolerance ends up with an effect resembling what Guttman (2000) called “the labelling dilemma”, i.e., by describing people as ill or vulnerable they are inadvertently labelled as such, which may paradoxically confirm their stigma despite the intervention’s intention to achieve the exact opposite.
- Achieving understanding without the need to question existing power hierarchies: The idea of pedagogy of tolerance is that those with the power to stigmatize (and execute a *power over* others) may or may not tolerate the stereotype of “stigmatized people” in a paternalistic top-down fashion due to an elicited empathic understanding. This aimed-for empathy may, however, very well be a form of sympathy as the Other usually is depicted as problematic and/or suffering. In doing so, the idea of “helping” with its implicit hierarchical relation between the helping and the helped is evoked. Bromseth and Darj (2010) add that this aspect’s impact is often intensified by missing that a member of the Othered group may be part of the normative majority being educated which re/produces the separation between “us” (the normative majority) and “them” (the Othered minority). Moreover, the image of the Other as “the problem” remains intact and dismisses the need to question and investigate the tolerating majority’s own contributions to the existence of the stereotypic images about the Other. This lack of self-reflection supports the maintenance of a clear distinction between the problematic Other as inferior and the non- and unproblematic moral majority as superior.
- Achieving understanding while maintaining the vulnerability of the Other: “The stigmatized” as the receiver of understanding/help have to deal with whatever reactions may be elicited, during and after the intervention. Due to this, the situation of the Other remains vulnerable, turning contributing to tolerance-targeting interventions into a risk as the Other enters a stage on which s/he could meet all kinds of reactions from rejection to acknowledgement (see e.g., Berg, 2010). The diversity prophets are supposed to go to the mountain of normality to influence it. Doing so might, however, very well imply to beg those who actually represent the threat not to fear or suppress the threatened.

My intention with this brief debate article is to suggest and describe another way of dealing with the stereotypic expectations accompanying and creating stigma by targeting its normative basis. This strategy is called “norm-critics” or “norm-critical education” and can be understood as an alternative to pedagogy of tolerance. Consequently, the two approaches will be discussed comparatively. Before describing the theory and practice of norm-critical education, a word of explanation: the terms “majority” and “minority” are used not in a quantitative but in a normative way in this text, i.e., they denote theoretical collectives that either address “those who are considered normal” (majority) or “those who are considered deviant from the norm” (minority).

## Norm-critics’ background and approach to change

Educationalist Kevin Kumashiro (2002) suggests an anti-oppressive education strategy that is supposed to change students and society and fuses elements of queer theory and critical education. It has been termed “norm-critics” or “norm-critical education” in Sweden (Bromseth & Darf, 2010). On the one hand, norm-critics include queer theory’s emphasis on analysing and destabilizing societal norms as a result of cultural processes, based on a post-structural view of reality with binary-coded, hierarchical pairs of concepts (such as health—disease) which determine what is regarded as both opposite and by comparison superior/inferior concepts. Moreover, norm-critics is based on and applies an understanding of power as productive in a Foucauldian sense, a *power through* concepts and ideas constructing reality which is enacted by everybody, not only by those with superior *power over* other people (Jagose, 1996; see also Spencer’s [2014] adaptation of Lukes’ [2005] analysis of different types of power). On the other hand, the queer approach is combined with aspects of critical education. It adopts its understandings of knowledge as never neutral and its main aim of raising a critical consciousness (conscientization) in order to counteract oppression as well as social exclusion and to change society (Freire, 1973). Raising a critical consciousness is especially imperative for norm-critics’ success, only this time the normative majority’s understanding of the world and its consequences are in focus, not that of the Other. Based on this understanding, transformative social actions can be promoted and developed by the normative majority (cf. Matthews, 2014).

According to Bromseth and Darj (2010), norm-critics target the normative majority’s understanding of the world by uncovering norms with everyday significance, i.e., the often unquestioned and unconsciously applied normative rules, assumptions, and expectations regarding how to think and act. By exploring the normative content and impact of those expectations and images, norm-critical education allows for an understanding of one’s own actual and potential contribution to the features of reality and raises awareness for the privileges which members of the normative majority enjoy. It should, however, be noted that calling a rule, an assumption, and expectation “a norm” is often misunderstood as calling them “a bad thing”. But that is not necessarily the case because norms are necessary and unavoidable as they are needed to enable social contacts. There is simply no such thing as a normless society. What norm-critics advocate is not to discard all norms but to make all norms potential objects of investigation in order to understand who benefits from the norm and who loses because of its application and to open up a space for (possibly, if agreed upon) acting on this understanding in a way that broadens normative possibilities.

Examples for norms in the field of health are the performance norm (i.e., you have to regard good health as something which has to be actively enhanced), the potentiality norm (i.e., you should perceive good health as a prerequisite for having the chance to live a good, self-determined life), the feasibility norm (i.e., you should be convinced that good health can be created), or the presentation norm (i.e., you should have a slim, well-trained body in accordance with what is expected of the sex you have been assigned at birth) (Pelters, 2012). Examples of how to work norm-critically with those norms are given in the section “How to do norm-critics”. These norms define the stereotypic image of a health-conscious person by codifying what deeds, looks, and ways of thinking are to be expected from such a person (cf. Brade, 2008)—and what constitutes “the health deviant Other” as the opposite of that health-conscious person. In doing so, a moral line between right = good and wrong = bad is drawn which includes a hierarchy of valued or rejected ways of being and acting as personalized in the figures of “the healthy norm person” and “the health deviant Other” respectively. Norm-critics represent thus a tool “to help make value-laden assumptions more explicit and to identify ethical concerns” (Guttman, 2000, p. 221), both applied to everyday life and in a self-reflective way to norm-critical interventions.

The concern of norm-critics is thus not with behaviour as such but with expectations about people exhibiting a behaviour, a bodily feature or other symbol understood as a normative health marker. Norm-critics does not aim at embracing deviant behaviours but at diminishing moralizing judgements concerning the people exhibiting certain behaviours and in doing so counteracting everyday shaming and blaming. This applies even if “the Other” does not develop an identity based on “health” as the person may very well be identified as a stereotypic figure due to normative expectations and suffer the consequences of being Othered despite the lack of this kind of identity. The practice of norm-critical education is hence focused upon raising awareness for the processes by which members of a normative majority re/construct images of stereotypic figures (such as “the fatso” or “the couch-potato”) with certain personal character traits which are to be condemned and in doing so limit the acting space of those identified as examples of those figures. An example of such a limitation has been shown by Mensinger and Meadows (2017) who observed that people with internalized weight stigma (IWS) develop less engagement in physical activity compared to an overweight group without internalized stigma. This led the authors to conclude: “Healthy living programs may be less effective for those most vulnerable unless we aim to reduce IWS” (Mensinger and Meadows, 2017, p. 64; see also Nolan & Eshleman, 2016).

Normative assumptions and expectations are moreover regarded as constituent parts of a coherent narrative of power (as e.g., heteronormativity) which represents the very basis for the functioning of these normative assumptions and expectations. In other words: norms are always related to questions of power as they represent and realize what I have described as *power through* (see above). In the health context, healthism according to Crawford (1980, 2006) could be such a narrative. Bringing norms to our attention provides an opportunity for scrutinizing and questioning their eligibility, debating for whom they are and are not useful or beneficial, what aspects of reality emerge and which are kept back, and what consequences they implicate, not only for the deviant but for everybody. By raising critical consciousness in members of the “normal” moral majority for how they produce the self-evidently seeming, stigmatizing, and excluding norms and what consequences they imply, norm-critics focus on inducing change in those who stigmatize instead of being stigmatized, those affecting not affected. Thus, those who are considered responsible for adverse influences on minorities and powerful enough to direct opinions are put in charge for change. In other words: instead of asking diversity prophets to go to the mountain, it is the mountain of normality that we should be seeking to move.

Using Guttmann’s (2000) value-centred analytic approach as an inspiration for understanding norm-critical interventions, the idea of norm-critical education can be summarized as follows: norm-critical education starts off with regarding the joined construction of a problematic reality (here: concerning stigma, blame, and shame) as the problem in need of solving. The solution (and intervention content) is to raise a critical consciousness for norms, for everybody’s involvement in the process of constructing that specific normality, and for the consequences for the Othered group as well as to question the eligibility of these norms and to work towards change. Norm-critical education is thus an approach that encourages self-reflection, critical thinking, and emancipation.

## Why norm-critics instead of pedagogy of tolerance in health education?

First a little disclaimer: I have discussed “pure versions” of both norm-critical education and pedagogy of tolerance in this section. It should be noted that there may be interventions with mixed perspectives that achieve good results in making people reflect and raise awareness. Having said this, let me discuss how norm-critical education may deal with pedagogy of tolerance’s mentioned problems (stereotyping; supporting existing power hierarchies; maintaining the vulnerability of the Other), which remain despite this pedagogy’s general potential to mitigate the problematics of stigma and blame.

*Stereotyping*: The problem of stigma, (self-)blame, guilt, or even helplessness and frustration experienced by “the Other” is to a great extent situated and grounded in evaluating and being evaluated on the basis of value-laden stereotypes, i.e., by turning to normative images and expectations to label people as “health-deviant” and, in doing so, assuming that those people are marked by flaws of character and a lack of virtue accompanying health-deviance (Crawford, 2006; Guttman & Salmon, 2004). To raise awareness for these normative and limiting expectations and images is a focus of norm-critics, not of pedagogy of tolerance. On the contrary, pedagogy of tolerance needs to work with stereotypes in order to describe and distinguish a specific group as a defined “something” which tolerance then can be executed upon. Therefore, it inevitably yet probably inadvertently confirms stereotypic norms.

To compare the two strategies and clarify the different approaches, the example of the “fatso” is considered. In pedagogy of tolerance you could have an overweight person talking about how it is to live with overweight, different reasons for becoming overweight (like diseases or living conditions, apart from nutrition), the—usual—disadvantages or downsides which “fat or overweight people” experience because of their overweight (instead of advantages or upsides which also might exist), and probably how “fat or overweight people” want to be treated. The whole exercise aims at eliciting empathy and understanding for a situation which is in all probability presented as a complicated one (you usually don’t invest money in raising empathy for rich white men, even if their life could be difficult). This is usually done without or with only little probing of participants’ expectations and assumptions.

The norm-critical way could start by asking for images of “the fatso”, what expectations there are in the group regarding e.g., the reasons for being fat, that person’s employment situation, hobbies, family/partnership, friends, or other aspects of that person’s everyday life. You could add questions about the expected gender, class background, ethnicity, functionality, etc. If you do this in small groups, the results of the different groups can be compared. Very often, these results tend to be quite similar or may at least allow for locating some types or tendencies. These results open up for asking about:

- Ideas about character traits which a person like that could have, thereby eliciting common stigmatizing normative assumptions (see e.g., Latner & Stunkard, 2003);
- The reasons for the probably occurring similar images, thus making the connection between the individual and collective normative thinking in the group and in society at large;
- Probable consequences for how people regarded as “fatsos” will be treated in different situations (for an example on job-seeking, see Powroznik, 2017) and for how people who want to avoid being regarded as “fatsos” treat themselves, with the aim of problematizing the privileges of being considered “thin”;
- The correctness of these images, assumptions, and expectations, e.g., by comparing the result of the discussion to famous or familiar overweight people and their lives, thus intentionally questioning and broadening existing views with the help of a “reality check”.

From there on, these views can be problematized and new ways of thinking about overweight people can be invented and tested in the group. Last but not least, ideas about what actions to take to work towards a change of these for a lot of overweight people probably very problematic normative assumptions can be developed. Norm-critical education is thus a matter of consciously constructing and deconstructing norms.

As shown, the focus on norms turns the norm-critical strategy into (a) a more profound one when aiming at change (investigating basic processes instead of presenting ready-made images) despite the usual risk of education of never being able to anticipate what results will emerge from an intervention, and (b) escapes the risk of consolidating rather than challenging norms as the actual norms in the group are a direct focus of inspection, questioning and development (cf. RFSL Ungdom and Forum för levande historia, 2011).

*Supporting existing power hierarchies*: In pedagogy of tolerance actors may easily adhere to an interpretation of the difference between their own position and the position of the subordinate/deviant that allows for secluding oneself from the position of the subordinate by assuming the position of a “good authority”. Hence, neither the hierarchical inferiority of unhealthy minorities nor its image as “a problem” changes fundamentally. Blame and stigmatization may therefore always resurface in new, potentially challenging ways and may thus still present a barrier to health-deviant minorities’ health development, as e.g., the literature on advocating personal responsibility has been pointing out repeatedly (Guttman & Ressler, 2001; Ten Have, de Beaufort, Teixeira, Mackenbach, & van der Heide, 2011). In contrast, norm-critical education does not confirm and maintain the image of the Other as “a problem” as it deals with a completely different problem: the co-construction of a certain reality, the consequences this reality may have, and their eligibility.

Due to the rather abstract nature of its interventions (providing personally unconnected, even unchallenging knowledge about “the Other”), pedagogy of tolerance appears even as not sufficiently far-reaching. In norm-critical education the crucial process of raising a critical consciousness regarding one’s own contribution to reality is in contrast much more linked to personal experiences and reflections of everyday significance. It is thus supposed to have a deeper, more personal impact and seems more likely to succeed in permanent change, compared to pedagogy of tolerance, as it makes a different kind of threshold knowledge accessible. The deeper impact becomes even more probable by the two following steps, both of which resemble critical pedagogy in the wake of Freire (1973), i.e., reflecting on the consequences of norms and the normative spectrum and, finally, developing plans for action (cf. Matthews, 2014).

Moreover, norm-critics’ understanding of power as productive (*power through*) rather than oppressive may support the success of interventions which are not based on processes of othering and the execution of a top-down *power over*. This constellation may buffer the resistance to health messages induced as a trial to protect one’s identity against shame and social exclusion (Blaxter, 1997; Broom, 2008; Whitehead & Russell, 2004). The effect could be enhanced even more by appreciating the situated cultural significance and emotional importance of practices usually labelled as “risk behaviours” (such as smoking) as a sign of respect for (relational) autonomy (cf. Guttman & Salmon, 2004). Such interventions may also counteract shame as a major barrier to empowerment as well as to facilitating/enabling personal change (Brown, 2006). Whereas shaming may counteract the success of the mentioned anti-stigmatization coping interventions, it could be made aware and be reflected upon in a norm-critical conscientization process—as could be the mentioned “risk behaviours” as representations of quality of life or cultural accomplishments, in particular socio-ecological milieus (Guttman & Salmon, 2004).

*Maintaining the vulnerability of the Other*: Whereas pedagogy of tolerance supports the image of the Other as inferior as well as problematic and prescribes the Other as a vulnerable figure, norm-critical education rather reveals such labels. Being subjected to the normative majority’s judgement will also be avoided in norm-critics as nobody has to come out as a representative of “the Other” in norm-critical education, in contrast to (many) tolerance-focused interventions, which limits the risks of vulnerability considerably.

Norm-critical education is often presented as inclusive regrading both representatives of “normality” and “abnormality”. The inclusive, equalizing approach is supposed to break up the vulnerable position of the Other and may support a balancing of power. The inclusive approach is understood as another aspect in favour of norm-critics in contrast to pedagogy of tolerance which has been criticized for implicitly presuming that its participants all belong to the normal and normative majority (cf. Brade, 2008). However, the approach seems to contradict norm-critics’ focus on the normative majority as target group. To deal with this situation and stand a chance to keep the inclusive approach, it is necessary to keep the following in mind: on the one hand, “normal” and “abnormal” people are assumed to work together to uncover and question the norms of the moral majority. It is thus not the deviant minority who is doing the work, bears the responsibility for change, and is in the limelight. On the other hand, the openness for co-operation is a balancing act considering commitment, responsibility, and attention as it is always easier to focus on deviations than normality with its usually self-evident, implicit, and unquestionable character.

The most convincing argument, however, is still the following: coping lays the burden of taking responsibility for change on health-deviant minorities, i.e., on those that are considered as being in a less powerful position, by targeting them as agents of change. Pedagogy of tolerance might also lay this responsibility burden on health-deviant minorities as these interventions often are conducted by members of these minorities, i.e., they become responsible for changing others, not themselves. Norm-critics, in contrast, answers the dilemma of whom to target in this kind of intervention (Guttman, 2000) differently and holds those directly and personally liable who actually stigmatize and label people as “deviant” and thus puts in charge of change on a societal level those who have been in charge for the problem in the first place. To engage in norm-critical activities is regarded a way of showing solidarity, of bonding and kinship, by considering and acting upon one’s responsibility for social circumstances that may complicate people’s lives (Guttman & Salmon, 2004).

It can be concluded that norm-critical education represents a different way of conceptualizing and thinking about health education when compared to pedagogy of tolerance (cf. RFSL Ungdom and Forum för levande historia, 2011). As the aimed-for change is supposed to concern the societal level, norm-critical education as a “package” of different characteristics is even considered a new approach in health education altogether as it is directed at normative thinking and its consequences (instead of behaviours), linking personal to societal change (instead of focusing on one’s direct environment), getting participants personally involved (instead of thinking about social determinants of health on an abstract level), and targeting the dominant normative majority (instead of inferior and stigmatized Others).

Ultimately, norm-critical education in the wake of Freire (1973) aims at changing the acting space for everybody in society, not only for “the healthy-deviant Other”, as all our possibilities of being health-aware and responsible agents and citizens are limited by norms which always include the potential of turning someone into “the deviant Other”. This stigmatizing potential, in combination with the opposing capitalistic demands of control and pleasure in need of juggling (Crawford, 2000) and the importance of a moralized health (Lupton, 1995), poses a challenge that seems to be met with more and more uneasiness in society (Pelters, 2017) and has been increasingly associated with the term “health craze” (see e.g., Sundström, 2015) in Sweden. As changing the acting space of the members of society means changing society, norm-critics may moreover contribute to mitigating what Guttman (2000) called the distraction dilemma by reducing the role of health in society and thus freeing capacities to work on important social issues which have been distracted by health craze. The importance of health in conjunction with the emergence of a critical voice in the health discourse may provide good conditions for testing norm-critical education.

As all intervention strategies have their downsides (Guttman, 2000), so does of course even norm-critical education. Before I turn to the practice of norm-critical education, I want therefore to conclude this section by mentioning its two biggest concerns as experienced by norm-critical educators such as J. Bromseth (mentioned in personal communication, November 30, 2013): first, realizing one’s contribution to the co-construction of a problematic reality may induce some sort of personal crisis as a possible discrepancy between one’s self-image as unprejudiced and one’s actual way of thinking in stereotypic ways might be self-reflectively realized. Second, the lack of given information may be perceived as insufficient in certain cases (e.g., when knowledge of the path of infection may mitigate reactions to infected people). Using norm-critical education should thus necessarily be linked to an evaluation of the situation and to certain requirements on the part of those leading norm-critical interventions.

## How to do norm-critics

Doing norm-critical education requires a sound knowledge base regarding the definition and function of norms in general and regarding health in particular, as well as the intersection with other orders of power (gender, ethnicity, age, etc.). The intimate connection between norms, questions of power, and privileges should be understood. Moreover, a personal awareness about one’s own position in society and one’s involvement in the re/production of norms should be achieved by self-reflection. It is imperative to understand how norms (and we who apply them) create and shape the conditions for discrimination, stigmatization, marginalization, and inequalities, but also that this is work in progress—norms can and have changed in time and place and it is change which norm-critics aims at: ‘The goal of [norm-critical methods] is that both leaders and participants should detect, reflect upon, and get tools to change values that one has and norms that prevail in one’s surroundings’ (RFSL Ungdom & Forum för levande historia, 2011, p. 14).

There are a number of norm-critical “method books” and collections of norm-critical exercises available (e.g., Brade, 2008; RFSL Ungdom, 2011) but these focus, for the most part, on norms regarding sexuality and gender. Being a norm-critical health educator demands thus a certain amount of creativity in order to adjust existing exercises, which originally target different orders of power, or to invent new exercises. In order to conduct norm-critical educational activities in practice, it is furthermore desirable to participate in various norm-critical exercises to first get a feeling for the approach in order to become capable of deliberately creating a respectful and open atmosphere that promotes dialogical discussions. Educators are recommended to have access to more experienced mentors who support one’s familiarization with the approach but, most importantly, educators should never stop understanding themselves as learners instead of masters (Brade, 2008; RFSL Ungdom and Forum för levande historia, 2011). Freire’s pedagogical influence can be easily recognized here (Freire, 1973).

Giving norm-critics’ focus on discussing and raising awareness, Brade (2008) recommends conducting norm-critical education with adolescents and adults aged 12–13 and above but suggests that it may even work with younger children if the methods are properly adjusted, albeit target group adjustment is an obvious demand regarding all participant groups. One ground rule for a norm-critical education is then that especially the (seemingly) “normal” members of a group need to be involved to engage those so-called “normal common people” in reflecting on norms and raising awareness for the normative processes and their consequences in which they themselves are involved. In doing so, those who are deemed to be powerful enough to reproduce but also change normative assumptions and expectations are expected to be reached. The norm-critical approach has to this day most commonly been used in different types of schools as institutions of formal education as well as in youth organizations, both as an educational activity and as a cornerstone in structural equal opportunities promotion and diversity work (see e.g., Bromseth & Darj, 2010; Martinsson & Reimers, 2008). RFSL Ungdom and Forum för levande historia (2011) point out, however, that this approach could easily be used in working life and other institutional contexts as well.

As normative judgments are often made in the blink of an eye, based only on people's appearance, visual methods appear to provide an appropriate way to uncover norms. Let me give three examples. *Gradually changing a presentation to uncover expectations*: in a series of images, exercising women with different bodies are shown and the question is raised why these women supposedly work out. The first image shows a middle-aged, white, able-bodied woman with a body mass index (BMI) within the recommended weight range. Here, the students might claim that she is possibly working out for fitness reasons. The second image shows the same type of woman, yet with the exception of her being chubby. Now, the students might expect her to train to lose weight. In the last image, a middle-aged, able-bodied, chubby woman is shown who is not Caucasian and wears a hijab and a dress. This might pose a considerable problem for the students who might have a hard time imagining why that woman is training. Finally, they might consider her doing a test-exercise and might suggest that she is actually not really training at all. Next, it would be possible to carve out the involved and intersecting norms and consider how these expectations may guide how the respective woman may be treated, what consequences the students’ normative expectation thus may have. Another example is *working with ridiculing opposites*: Austrian artists deliberately played with expectations by turning reality upside down and present a video titled “abs, legs and fries” instead of “abs, legs and thighs” (as the name is a wordplay in German, this is no literal translation but one that captures the spirit of the wordplay). This is a persiflage on exercise videos which uses the same dramatic effects and style but propagates for “unhealthy” or ridiculous exercises instead. The video could be used to discuss why we laugh when we watch it or what expectations we have and in what way they are counteracted by the clip in order to uncover norms on exercise and diet. As norms are always related to power, the last suggested exercise targets the derogative, disempowering consequences of being considered unhealthy or health-deviant directly: *discussing privileges* of those who successfully present themselves as healthy. Similar exercises have been made regarding heterosexual privileges in interventions aiming at reducing sexual minority stress (Chaudoir, Wang, & Pachankis, 2017).

Some of these methods can be used to work with the four health norms mentioned earlier, the norms of performance, potentiality, feasibility, and presentation. To take on those norms, it would be possible to use working with ridiculing opposites as a discussion prompt. Here, the educator could present a short movie in which a young, slim, well-trained woman is shown in four situations: on a job interview, on a date, when attending a physician, and at a gym. But instead of presenting her as attractive and similar to other people she meets, the other characters represent another norm in the movie, namely a type we have learned to consider as e.g., fat, not-so-attractive, and/or disabled. It should also become clear that another health regimen is followed in this fictional reality. It is important that the movie shows the deviance of the woman by the looks, comments, and other behaviours of the majority which her appearance elicits so that the limitations which she experiences become clear. For example, her date could look disappointed when she enters and fake a phone call to end the date as soon as possible; the people doing the job interview could ask questions in an insulting way, implying that this is a waste of time, and leaving the woman more and more desperate. The physician could talk about her bad health caused by this bad, physically active lifestyle of hers and her possibility of letting health just “naturally” come to her, in a doctor’s office decorated by art prints of hamburgers and hammocks. On top of it, the gym could be hidden in a dark back road with a doorbell, a secret password, and a bunch of “health freaks” who shamefacedly indulge in their addictive and vile workouts, labelling it as a secretive, unwanted if not forbidden activity. Usually movies of that kind cause laughter or other emotional reactions because they generally appear completely ridiculous to our biomedicalized gaze which functions as a clue to bring forth normative thinking.

The discussion could then follow the above-mentioned path and focus on the question why we laugh (or have that particular emotional reaction) when we watch it, what expectations we have regarding health and the person who looks and/or behaves in a certain way, and in what way they are counteracted by the clip. This is done in order to uncover the above-mentioned norms as well as our knowledge about the norms, implying our general complicity considering their reconstruction. In the next step, privileges of those considered as healthy can be discussed, like e.g., having a higher probability to get the good “fancy” jobs or partners as well as appreciation and attention because of their commitment to maximize health. Here, the often-appearing objection “but isn’t that norm a good thing?” may be dealt with by collectively reflecting upon which people will experience a norm-derived benefit and which people will not do so. The latter group may include people with family and several jobs resulting in a lack of free time to engage in physical activity, thus not complying with the performance norm; people who, due to their body size, constantly get commentaries about starting to exercise despite the fact that they already do so, thus not complying with the presentation norm; people getting no promotion in an “ambitious” environment like a law firm but a lot of funny looks due to their body size, thus not complying with the potentiality norm; or, finally, people who have lived according to the so-called healthy lifestyle and got cancer anyway, thus not complying with the feasibility norm. A conclusion could be that whatever you do, it may not be enough (concerning feasibility), or there may be other factors which you cannot control that limit your possibilities (concerning presentation), or there do not have to be inescapable consequences of behaviours (concerning potentiality and performance). These possible conclusions are thought to question the connection between the norms and expected outcomes in order to show that there are no simply, inescapable “either/ors” here.

It is possible to continue by negotiating how all of these consequences and privileges resonate with our ideas about a good life and the moral or ethical principles we consider valuable for treating people and for how we want to be treated ourselves, like anti-discrimination laws, the principle of justice, expectations regarding good citizenship, the right of self-determination, etc. Finally, the question of what to do with the addressed norms can be considered, hopefully leading to some practical implications for everyday practice in the group and for the individual we have been working with. One opportunity would be to modify the norms in a relativizing way by being more specific and/or by adding “to a certain extent” to them to allow for other ways of achieving good health and of being healthy. For example, could the performance and the feasibility norm be modified to “You should regard good mental and physical health as something which can be actively enhanced to a certain extent” and “You should be convinced that good health can be created to a certain extent.” Another imaginable way of dealing with the creative challenge of modifying norms could be to add a norm demanding an effort for understanding, such as “Whatever you think first of a person’s health [or more specifically: health-related looks], you should consider it preliminary and seek to understand this person’s health (actions, notions etc.) in the context of this person’s living conditions.” Then, of course, you could try to change a norm completely, e.g., the potentiality norm from the original “You should perceive good health as a prerequisite for having the chance to live a good, self-determined life” to “You should perceive the chance to live a good, self-determined life as independent from your health.” But as norms are social rules of conduct in communities, they of course need to be collectively negotiated so that the mentioned scenario and modifications are only hypothetical suggestions. The basic idea is not to advocate “anything goes” but to advise caution and create an opening for a contextualized understanding of other people and for reflecting our way of thinking and acting.

## A task for education?

Last but not least, the question remains whether or not pedagogical approaches are the only or best ones for accomplishing increased understanding of social norms and their contribution to inequalities. Ostensibly, and as the question already implies, pedagogy is not the only way to raise awareness. There are other possibilities such as works of art or literature (e.g., the art collection “Equity is the answer” [n.d.]), awareness-raising campaigns in the media (see e.g., ELINET, 2015) or even implemented laws and regulations (such as the Swedish Discrimination Act, 2008, p. 567) which intend to call to attention and promote relevant questions and processes. Due to this article’s limited space, I will contemplate only these three possibilities. I will do so assuming that they are different from “pedagogy” in a narrow sense as consciously initiated learning events, well aware that being exposed to all three of them may initiate processes of learning which may turn them into (potential) educational devices and thus a part of a broader defined “pedagogy” including implicit learning.

Both common regulations and awareness campaigns share the feature of being able to reach a lot more people quicker and in a more aligned way than educational activities. These are major flaws of education which may be met to a certain degree by compulsorily implementing educational content for all, e.g., in a comprehensive context (e.g., at school). The advantage of education, however, is that —if conducted in the suggested way—it implements a bottom-up dialogic instead of a top-down (semi-)paternalistic way of eliciting understanding which may be linked to a number of ethical dilemmas (Guttman, 2000). To use Freire’s terminology (1973), an opening for problem-posing instead of banking-style education is created. That may induce a more embedded, deeper learning that is related to people’s own experiences and value-systems. In contrast to regulations and campaigns, works of art and literature may initiate a more thorough contemplation process directed at exploring one’s attitudes and thereby normative expectations concerning a certain phenomenon. However, engaging in that activity may not necessarily lead to awareness of one’s privileges, let alone to change connected to a re-evaluation of one’s role and its induced consequences. People may be more inclined to confirm and/or defend their normality as long as possible if not invited to broaden their perspective in a respectful and secure, yet challenging, dialogue.

Being able to provide this context is, in my opinion, the most powerful advantage that a well-executed pedagogical activity has to offer and the reason why I deem pedagogical approaches in general and the presented pedagogical approach in particular as, to date, the most promising one for accomplishing increased and personalized understanding of social norms and their contribution to inequalities as well as for working towards change. It may take some time to remove the mountain but I think that this is time well spent.

## Conclusion

Norm-critical education is a way of making members of the (presumably healthy) normative majority uncover and question their health-related norms. This is deemed valuable for holding those responsible for a change of attitudes towards health-deviant, disadvantaged minorities, who are the source of stigmatizing tendencies and own the power to work in an anti-oppressive and inclusive way. It is concluded that norm-critics render a valuable and much needed addition to the health intervention repertoire especially in the field of health disparities which to date has mostly focused on coping and applying pedagogy of tolerance.
